# Emergency health care in crises

**DOI:** 10.2471/BLT.24.020124

**Published:** 2024-01-01

## Abstract

Efforts to strengthen health systems to deliver emergency care are intensifying against the backdrop of increasing humanitarian crises. Fid Thompson reports.

When a series of earthquakes hit the western province of Herat, Afghanistan, on 7 October, an already struggling health system came under tremendous strain. “The quakes flattened whole villages in Herat province, leaving tens of thousands of people in urgent need of humanitarian and health assistance, including life-saving emergency care,” recalls Dr Alaa AbouZeid, World Health Organization (WHO) Team Lead for Emergencies in the country.

Within hours of the first quake, AbouZeid was working with colleagues from the International Committee of the Red Cross (ICRC), Médecins Sans Frontières (MSF), and other partners to support Herat regional hospital, helping with mass casualty management, treating the injured, and providing medicines and other medical supplies.

“We were able to rapidly mobilize resources at the most critical time,” says AbouZeid, noting that one reason for this was the preparedness of local health workers. “We had finished training them in the WHO/ICRC basic emergency care course, and putting in place a workable mass casualty management plan just a month before the earthquake hit. It made a huge difference.”

The Afghanistan earthquakes, which are reported to have killed more than 1400 people, were among the many emergencies that occurred in 2023. Ranging from natural disasters such as floods and droughts to armed conflicts, such emergencies often cause mass casualties, demanding rapid, effective emergency health-care provision.

WHO’s first guidance on essential trauma care was published in 2012, in collaboration with the International Association for the Surgery of Trauma and Surgical Intensive Care, but focus on the topic has sharpened since then, notably in the wake of the coronavirus disease 2019 (COVID-19) pandemic and as countries work on plans to improve pandemic preparedness.

“COVID-19 really brought home the need to boost and integrate emergency and critical care,” says Emilie Calvello Hynes, a technical officer in WHO’s department of Integrated Health Services. “Patients in respiratory distress had to be resuscitated and treated quickly to avoid respiratory failure and the need for mechanical ventilation. Many countries fell short, including high-income countries, but it was lower-income countries, particularly those lacking first-contact care capacity, and basic commodities such as medical grade oxygen, that were most vulnerable.”

WHO is currently working to address such shortcomings, partnering with 56 countries to establish strategic priorities and national road maps to strengthen emergency care delivery. “We need to prepare everyday health systems to be able to surge in large-scale emergencies,” says Calvello Hynes.

“Over half of trauma deaths occur in the pre-hospital phase.”

Alaa AbouZeid

Common strategic priorities include the introduction of the WHO emergency care toolkit, which includes clinical training using the WHO/ICRC basic emergency care course, an open-access training course for health workers who manage acute illness and injury with limited resources.

An interagency integrated triage tool is also being introduced along with the WHO emergency care checklist. This checklist recommends a review of actions at critical points to make sure that no life-threatening conditions are missed. WHO also recommends that countries improve emergency and trauma registries, to record facility data on acute illness and injury outcomes to help identify gaps in care.

WHO supported implementation of the emergency care toolkit to assist Afghanistan’s response to the earthquake in October. WHO and the Afghanistan health ministry have also been working on strengthening emergency and critical care capacity using doctors, nurses, managers and technicians trained in WHO/ICRC basic emergency care and WHO mass casualty management to train their peers across the country.

While pleased about the progress made, AbouZeid would like to see more done to improve pre-hospital services. “Up to 60% of trauma deaths occur in the pre-hospital phase, so we need to boost ambulance provision and get emergency trauma care units into smaller health facilities,” he says.

Dr Abdullah Al Azad, emergency officer for acute events in WHO’s Federal Republic of Somalia office, takes a similar view, arguing that ramping up robust pre-hospital emergency care delivered in primary health clinics is key to dealing with humanitarian crises, as well as the day-to-day emergencies faced by individual patients as a result of trauma, obstetric emergencies, and sudden complications of noncommunicable diseases such as asthma and cardiovascular disease.

According to Al Azad, the Federal Government of Somalia, working in collaboration with the World Bank and WHO, launched an initiative focused on improving the capacity of health-care professionals in Somalia to deliver emergency, critical and operative care services during the pandemic. “There was a recognition that there was an urgent need to improve care for critically ill patients,” he explains.

The overall goal of the project, which was implemented by the WHO country office of Somalia between 10 September 2020 and 31 March 2021, was to establish integrated service delivery by unifying and optimizing services in both primary care facilities and hospitals.

For Al Azad, it is important to see emergency health care as part of the whole system. “Effective emergency care, including pre-hospital care, is not an ‘extra.’ It is of fundamental importance and if it is not well managed, the effects are felt throughout the system,” he says. “The number of patient referrals goes up, as does the patient load at the district hospital and the central hospital. The result is that mortality and morbidity will increase along with financial costs to the system.”

The question of cost tends to loom large in discussions on emergency care, with the focus of attention on the perceived need for specialists and expensive medical equipment.

Calvello Hynes insists on the value for money that basic emergency care represents, citing a study published in the December 2022 issue of PLOS ONE that assessed the cost-effectiveness of implementing the WHO emergency care toolkit in two regional referral hospitals in Uganda.

The study showed that an initial investment of 5873 United States dollars (US$) in the two hospitals resulted in 34 lives saved, and the avoidance of US$ 1 670 689 in downstream societal costs. “The modelling of national scale-up indicated that over 5 years there would be a 655% return on investment,” Calvello Hynes points out.

“Strong emergency care systems […] are essential to preparing for the worst.”

Alegnta Gebreyesus

Having come through the harshest of real-world conditions, AbouZeid also highlights the cost-effectiveness of the WHO toolkit. “It contributes massively to saving lives with a few dollars for each patient. Supplies are not that expensive. Training is not expensive, and we cascade the training to the provincial level. So we're talking about a few thousand dollars per hospital,” he says.

WHO’s Clinical Services and Systems team is tracking mortality in Ethiopia, Nepal, Uganda and Zambia after these countries have implemented the WHO emergency care toolkit. According to Calvello Hynes, early indications show substantive improvements in patients’ outcomes.

Ethiopia has so far trained over 3000 health-care workers in basic emergency care. “We have very positive findings, especially with the improvement of process measures and early detection of critical illnesses,” says Dr Alegnta Gebreyesus, a trained emergency physician who is health diplomat at Ethiopia’s permanent mission to the United Nations.

Going forward, Calvello Hynes – like the other experts interviewed for this feature – would like to see attention focus on integration of the full spectrum of emergency, critical and operative care. “By some estimates up to five billion people lack access to safe and affordable surgical and anaesthesia care, and the inequity is stark,” she says, noting that 95% of people in the South-East Asia Region and sub-Saharan Africa in the African Region lack access, compared to just 5% of those in high-income countries. 

In May 2023, WHO Member States passed World Health Assembly resolution WHA76.2 advocating for “integrated emergency, critical and operative care for universal health coverage (UHC) and protection from health emergencies.” The landmark resolution, proposed by Ethiopia and Myanmar with over 80 co-sponsor Member States, passed unanimously and aims to address the burden of acute and critical illness around the globe.

While a clear indication of demand for emergency care within Member States, the resolution also makes explicit what, for people working in emergency care, has long been apparent: the UHC and emergency care agendas are intertwined. The strengthening of emergency services as a part of primary health care initiatives augments health systems’ capacity to respond to humanitarian crises, as much as to provide everyday emergency care to people.

By the same token, efforts to augment emergency care as a part of crisis response feed into efforts to achieve universal health coverage while also increasing preparedness. In an emergency situation, patient outcomes depend on how well the first-contact care system functions in the day-to-day provision of routine care. “COVID-19 taught us that preparedness matters,” says Gebreyesus. “And that strong emergency care systems rooted in primary health care are essential to preparing for the worst.”

**Figure:**
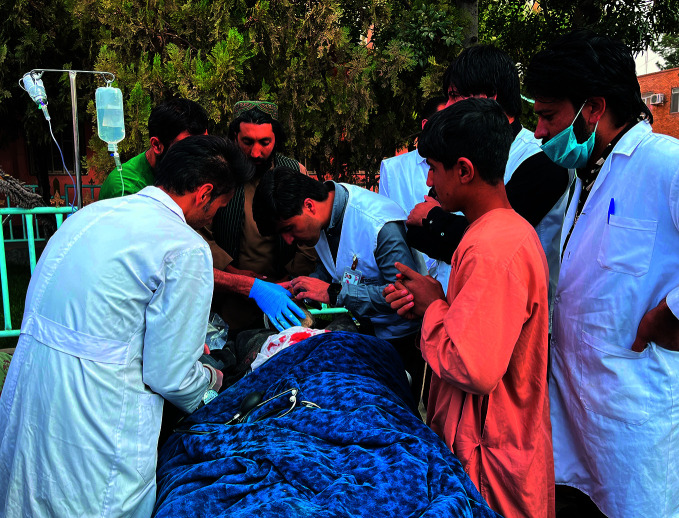
Medical staff attend to a patient at an emergency triage area in Herat, Afghanistan.

MSF/Paul Odongo

**Figure:**
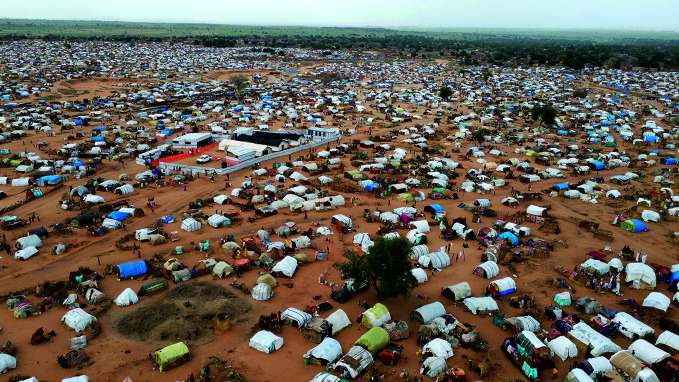
A health post in Adre, Chad, providing emergency health assistance to people fleeing conflict in neighbouring Sudan.

Courtesy of WHO Regional Office for Africa

